# A novel thermostable beetle luciferase based cytotoxicity assay

**DOI:** 10.1038/s41598-021-89404-z

**Published:** 2021-05-11

**Authors:** Sunju Choi, Hittu Matta, Ramakrishnan Gopalakrishnan, Venkatesh Natarajan, Songjie Gong, Alberto Jeronimo, Wei-Ying Kuo, Bryant Bravo, Preet M. Chaudhary

**Affiliations:** Jane Anne Nohl Division of Hematology and Center for the Study of Blood Diseases, Keck School of Medicine, University of Southern California, Los Angeles, CA USA

**Keywords:** Biological techniques, Cancer, Oncology

## Abstract

Cytotoxicity assays are essential for the testing and development of novel immunotherapies for the treatment of cancer. We recently described a novel cytotoxicity assay, termed the Matador assay, which was based on marine luciferases and their engineered derivatives. In this study, we describe the development of a new cytotoxicity assay termed ‘Matador-Glo assay’ which takes advantage of a thermostable variant of Click Beetle Luciferase (Luc146-1H2). Matador-Glo assay utilizes Luc146-1H2 and D-luciferin as the luciferase-substrate pair for luminescence detection. The assay involves ectopic over-expression of Luc146-1H2 in the cytosol of target cells of interest. Upon damage to the membrane integrity, the Luc146-1H2 is either released from the dead and dying cells or its activity is preferentially measured in dead and dying cells. We demonstrate that this assay is simple, fast, specific, sensitive, cost-efficient, and not labor-intensive. We further demonstrate that the Matador-Glo assay can be combined with the marine luciferase-based Matador assay to develop a dual luciferase assay for cell death detection. Finally, we demonstrate that the Luc146-1H2 expressing target cells can also be used for in vivo bioluminescence imaging applications.

## Introduction

Novel drug discovery approaches for the treatment of cancer depend on identifying therapeutics that can selectively get rid of diseased cells. These therapeutics include Chimeric Antigen Receptors (CARs), Bi-specific T-cell Engagers (BiTEs), Antibody–Drug Conjugates (ADCs), and small molecules. A sensitive and accurate cytotoxicity assay is essential for the successful identification and characterization of these therapeutics. Numerous assays are currently available to measure cytotoxicity. These assays utilize different detection methods such as radioactivity, fluorescence, colorimetry, and luminescence. Among cytotoxicity assays, the chromium release assay developed in 1968 is one of the oldest, and one that is widely used in research laboratories^1^. In this assay, Chromium-51 radionuclide (Cr^51^) is loaded into target cells, which is released into the supernatant upon cell lysis^2^. Besides the health risk of exposure to radioactive Cr^51^, this assay has many disadvantages including requirement of an expensive gamma counter and high costs associated with safe waste disposal. Further, the target cells have to be freshly prepared every time before the assay. Non-radioactive cytotoxicity assays were developed by loading target cells with a non-fluorescent molecule such as calcein-AM that is converted into fluorescent calcein inside the cells by the intracellular esterase activity. The release of fluorescent calcein into the supernatant upon membrane damage is measured using a fluorescence plate reader. CellTiter-Fluor is another fluorescence-based cytotoxicity assay with the mechanism-of-action similar to calcein-AM assay, although it measures the number of live cells. Even though fluorescence-based assays eliminate the use of radioactivity, they often have high levels of spontaneous release and variable cell labeling due to the differences in intracellular esterase activity^2^. Cytotoxicity assays that are based on the release of constitutively expressed cellular endogenous enzymes such as lactate dehydrogenase (LDH)^3^, glyceraldehyde 3-phosphate dehydrogenase (G3PDH)^4^ or adenylate kinase (AK)^5^ were also developed. However, these assays have poor sensitivity and do not distinguish between the death of target and effector cells in co-culture experiments. Flow cytometry-based cytotoxicity assays that are without any of the aforementioned disadvantages were developed^6^. However, flow-cytometry based assays usually involve careful calibration of the instrument and labor-intensive data analysis.

Bioluminescence occurs when an enzyme generally termed ‘luciferase’ oxidizes a light-producing substrate, e.g., luciferin or coelenterazine. Bioluminescence is naturally seen in a wide range of species, including bacteria, fungi, insects and marine organisms. Due to the sensitivity of luciferases to provide highly accurate read-out, they are one of the widely used reporters in biological studies^7^.

Luminescence-based cytotoxicity assays have been described (e.g., CellTiter-Glo), which measure cell death based on the amount of ATP present in the mixture. Recently, we developed an ATP-independent marine luciferase release-based cytotoxicity assay, termed Matador assay^8^. In the Matador assay, a marine luciferase of interest is expressed in the cytosol of target cells. The healthy target cells with intact membrane integrity retain the marine luciferase in the cytosol. However, the dead and dying cells with compromised membrane integrity release the marine luciferase, where its activity can be measured by addition of the substrate^8^. Furthermore, the luciferase substrate can also preferentially enter the cells with compromised membrane integrity and interact with the luciferase present in the cytosol. Although the Matador assay is highly sensitive and specific, marine luciferases are not ideal for in vivo bioluminescence imaging application due to the relatively high cost of their substrates (e.g., coelenterazine) and high background^9^. Therefore, cell lines engineered to express marine luciferases for their use in Matador assay cannot be utilized for in vivo bioluminescence experiments, thereby necessitating the need to generate two luciferase expressing stable cell lines; i.e., one expressing a marine luciferase for in vitro cytotoxicity assays and another expressing a firefly luciferase for in vivo bioluminescence applications.

Beetle luciferases are a class of luciferase with distinct chemical properties. Firefly luciferases are a specific subgroup of beetle luciferase^10^. Firefly luciferase (Fluc) is widely used for in vivo bioluminescence imaging applications^11^. However, past attempts at developing Fluc-release based cytotoxicity assays have failed due to its instability in tissue culture medium^12^ and lack of significant release of Fluc in the culture medium upon induction of cell death^13^.

Thermostable variants of beetle luciferases from *Photuris pennsylvanica (LucPpe)* have been described previously^10^. These luciferases were derived from *LucPpe* and have been optimized by a directed evolution based selection strategy that introduced as many as 34 mutations^10^. The mutants were selected for enhanced thermostability, signal stability (“glow response”), substrate usage efficiency and increased inhibitor resistance^10,14^. In contrast to marine luciferase, which produces “flash” luminescence, these mutants provide “glow-like” luminescence^10^.

To overcome the limitation of the short half-life of firefly luciferase, we developed a novel non-radioactive cytotoxicity assay, termed the Matador-Glo assay, based on the thermostable variant (Luc146-1H2) of beetle luciferase *LucPpe* as the reporter enzyme and D-luciferin as the substrate. We report that the assay is extremely sensitive, inexpensive, homogeneous, and can be combined with the marine luciferase-based Matador assay to develop a dual reporter assay. We further demonstrate that the cells engineered to express Luc146-1H2 can be successfully used for in vivo bioluminescence imaging.

## Results

### Development of a cytotoxicity assay using a thermostable luciferase that utilizes D-luciferin as a substrate

To develop a cytotoxicity assay based on a thermostable luciferase, we cloned the cDNA encoding Luc146-1H2, a thermostable mutant of beetle luciferase *LucPpe*, carrying a C-terminal Flag epitope tag into a lentiviral vector (pLenti-EF1α) in which the expression of Luc146-1H2 was driven by constitutive active human EF1α promoter. The vector also expressed the puromycin resistance gene (Pac) downstream of and in frame with the Luc146-1H2 cDNA and separated from the latter by a T2A ribosomal skip sequence. We also cloned the Luc146-1H2 cDNA into a second lentiviral vector (pLenti-CMV) co-expressing a blasticidin resistance gene.

**Figure 1:**
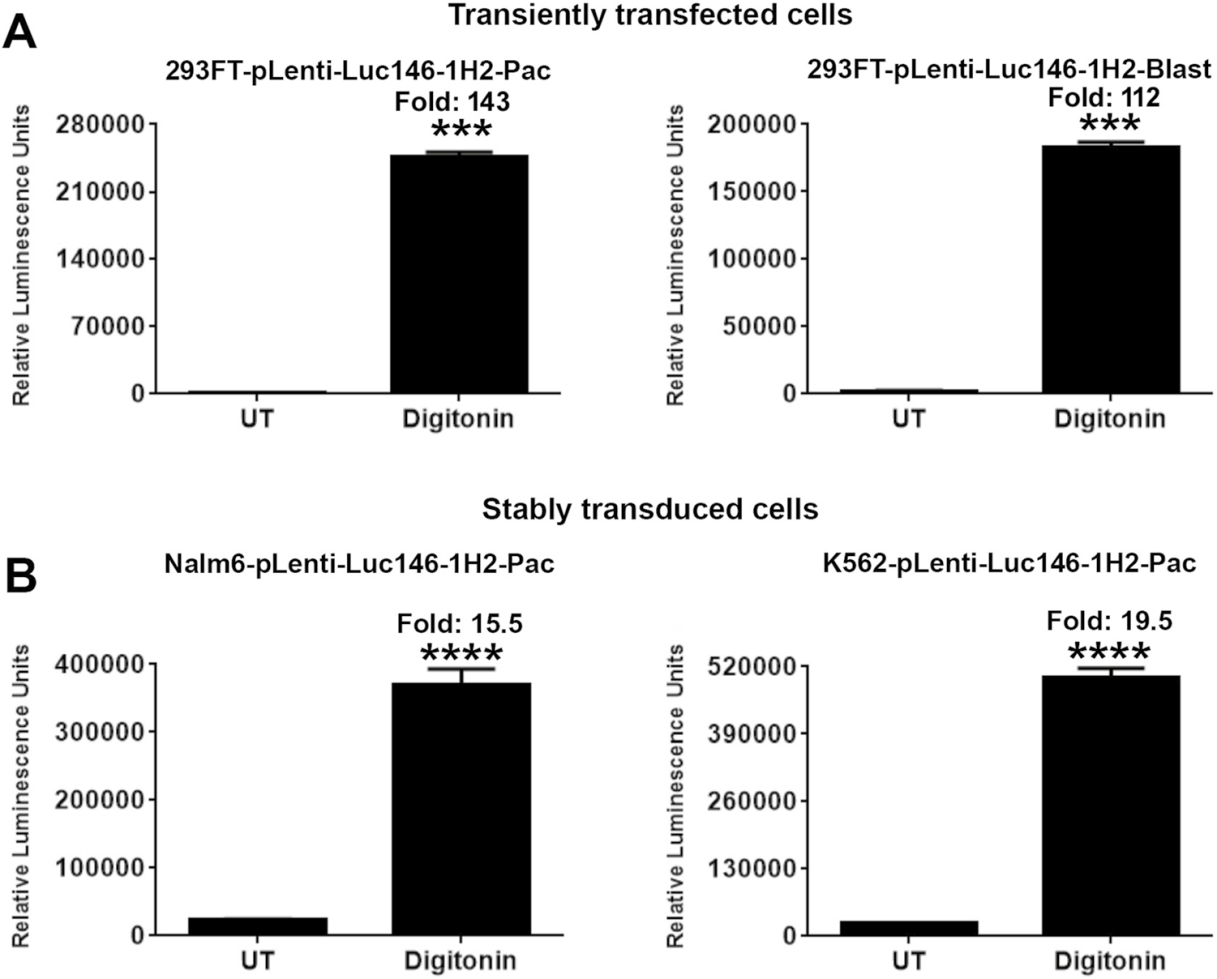
Validation of Matador-Glo assay using transiently transfected and stably transduced cells. (**A**) The indicated Luc146-1H2 lentiviral vectors were transiently transfected in 293FT cells. Approximately, 24 h post-transfection cells were treated with digitonin (30 µg/ml) for 90 min or left untreated (UT). Cell-free supernatants (25 µl) were assayed for luminescence by adding D-luciferin containing assay buffer (25 µl) directly to each well in a 384-well lumitrac plate. (**B**) Indicated cell lines stably expressing Luc146-1H2 were plated in a 384-well lumitrac plate and treated with digitonin (30 µg/ml) for 90 min or left untreated (UT). Luminescence was detected as described for (**A**)**.** The values shown are mean ± SE of a representative experiment performed in duplicate. Statistically significant differences are shown by asterisks (***) at a level of *P* < 0.001 and (****) at a level of *P* < 0.0001.

The constructs pLenti-Luc146-1H2-Pac and pLenti-Luc146-1H2-Blast were transiently transfected into 293FT cells. Twenty-four hours after transfection, cell death was induced by treatment with digitonin (30 µg/ml for 90 min) and the luminescence activity was measured by the addition of D-luciferin containing assay buffer. As shown in Fig. 1A, a marked increase in luminescence activity was seen in cells treated with digitonin in comparison to cells that were left untreated (UT). To test the assay on cells stably expressing Luc146-1H2, we infected Nalm6 and K562 cell lines with pLenti-Luc146-1H2-Pac virus. Stably transduced cells were selected with puromycin. Significant increases in luminescence were observed in Luc146-1H2-expressing Nalm6 (15.5 fold) and K562 cells (19.5 fold) that were treated with digitonin, as compared to the wells that were left untreated (Fig. 1B). These results indicate that a luciferase-release-based cytotoxicity assay (Matador-Glo assay) can be developed using cells stably or transiently expressing a thermostable luciferase such as Luc146-1H2.

### Matador-Glo assay is highly sensitive

**Figure 2:**
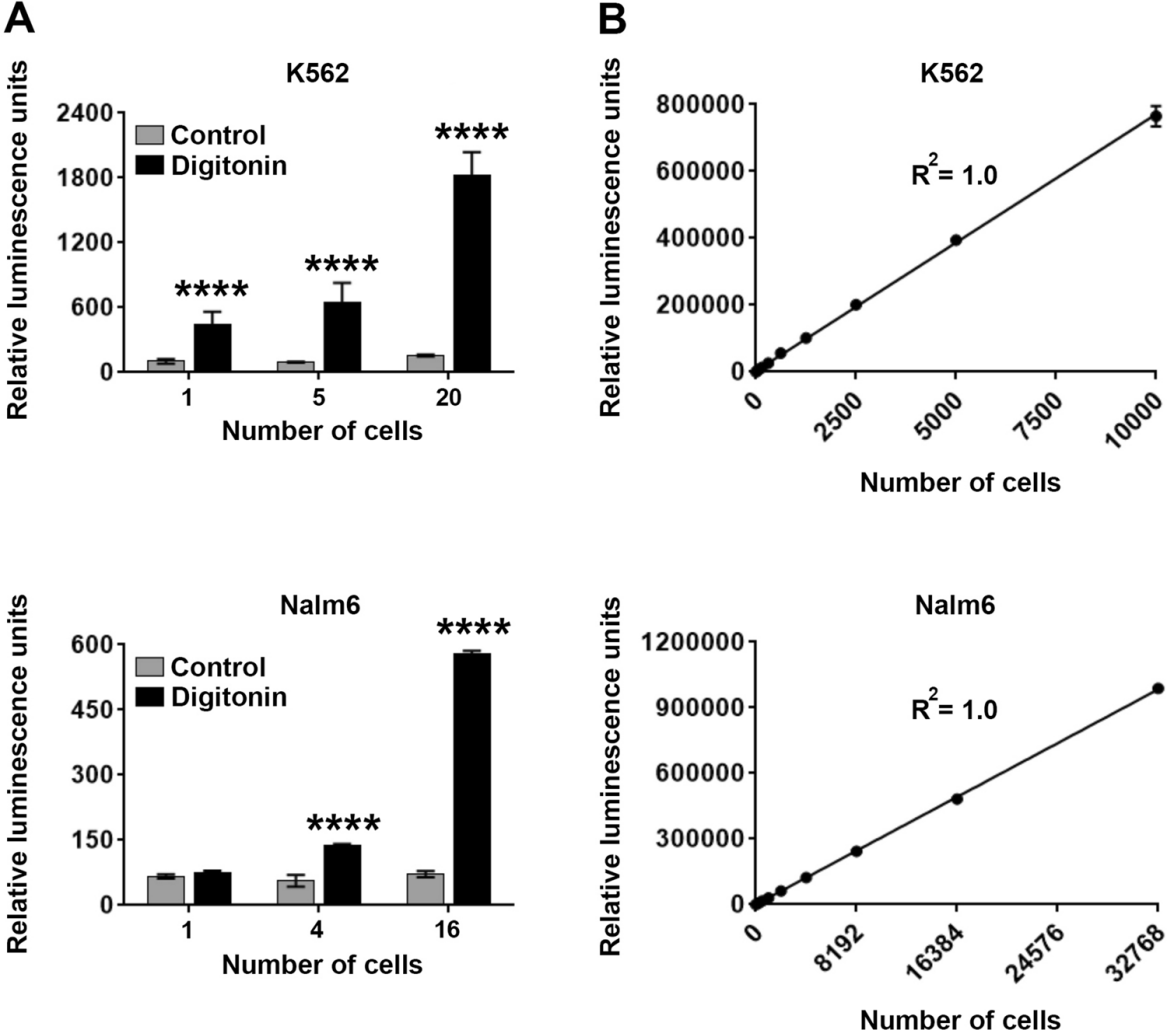
Matador-Glo assay is highly sensitive. (**A**) Indicated cell lines stably expressing Luc146-1H2 were plated in a 384-well at indicated numbers (by serial dilution) and treated with digitonin (30 µg/ml) for 90 min or PBS (control). Luminescence was detected by the addition of D-luciferin containing assay buffer directly to each well. (**B**) Linear increase in luminescence over a wide range of cell numbers in the Matador-Glo assay. Both the number of cells plated and luminescence values detected were plotted. R^2^ = Correlation coefficient. The values shown are mean ± SE of a representative experiment performed in triplicate. Statistically significant differences are shown by asterisks (****) at a level of *P* < 0.0001.

To check the sensitivity of the Matador-Glo assay, increasing numbers of K562 and Nalm6 cells stably expressing Luc146-1H2 were plated and cell death was induced by digitonin. A linear increase in luciferase activity proportional to the cell numbers was observed in wells treated with digitonin (Fig. 2A). Notably, a significant increase in luminescence was observed in wells that contained only a single K562 cell stably expressing Luc146-1H2 (Fig. 2A, top panel). The detection limit for Nalm6 cells stably expressing Luc146-1H2 was four cells (Fig. 2A, bottom panel). Furthermore, a perfect positive correlation between luminescence values and cell numbers was observed in both cell lines (R^2^ = 1) (Fig. 2B). Collectively, these results demonstrate the extreme sensitivity of the Matador-Glo assay for measurement of cytotoxicity.

### Matador-Glo assay can be performed either in cell homogenate or cell supernatant

**Figure 3:**
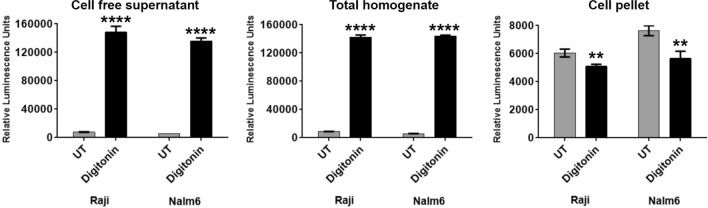
The Matador-Glo assay is a single-step homogenous assay. Raji/Nalm6 cells stably expressing Luc146-1H2 were either left untreated (UT) or treated with 30 μg/ml digitonin to induce cell death. Post-treatment, the cells were mixed well, collected in a 1.5 ml microfuge tubes and divided into 3 sets. Set 1 (cell-free supernatant), cells were centrifuged, and cell supernatants alone were collected in a new tube and plated in a 384-well plate, followed by measuring the luciferase activity. Set 2 (total homogenate), the cells with supernatant were directly assayed for luciferase activity by plating in a 384-well plate. For set 3 (cell pellet), cells were centrifuged, supernatant was removed, followed by the resuspension of cell pellets in PBS and plated in a 384-well plate to assess luciferase activity. Total reaction volume plated in 384-well plate from three sets was kept constant at 60 µl per well, and 15 µl of D-luciferin assay buffer was added in well mode to measure luciferase activity. The values shown are mean ± SE of a representative experiment performed in triplicate. Statistically significant differences are shown by asterisks (**) at a level of *P* < 0.01 and (****) at a level of *P* < 0.0001.

Ideally, a single-step homogenous assay is preferred to measure cytotoxicity. To check whether Matador-Glo assay can be performed in a single-step homogeneous manner, and to assess whether separation of cellular pellets from supernatants interferes with its sensitivity, we measured the luminescence from cell-free supernatant, homogeneous suspension (cells with culture medium), and in cells alone (pellet). For this purpose, Raji and Nalm6 cells stably expressing Luc146-1H2 were treated with digitonin, and the luciferase activity was measured in the above-mentioned fractions. As shown in Fig. 3, digitonin treatment resulted in a remarkable increase in luminescence activity in the fractions containing the cell-free supernatant and in homogenous cell suspension (total homogenate) and without any apparent differences in luminescence values between them. These results indicate that the Matador-Glo assay is a single-step homogeneous assay. A minor decrease in luminescence activity was observed in the cell alone (cell pellet) fraction of both Raji and Nalm6 cells stably expressing Luc146-1H2 that were treated with digitonin (Fig. 3). This could be due to the loss of Luc146-1H2 from cells that have lost the membrane integrity upon treatment with digitonin.

### Applications of Matador-Glo assay in testing CAR, Bispecific T cell engagers and Natural Killer cell-induced cytotoxicity

**Figure 4:**
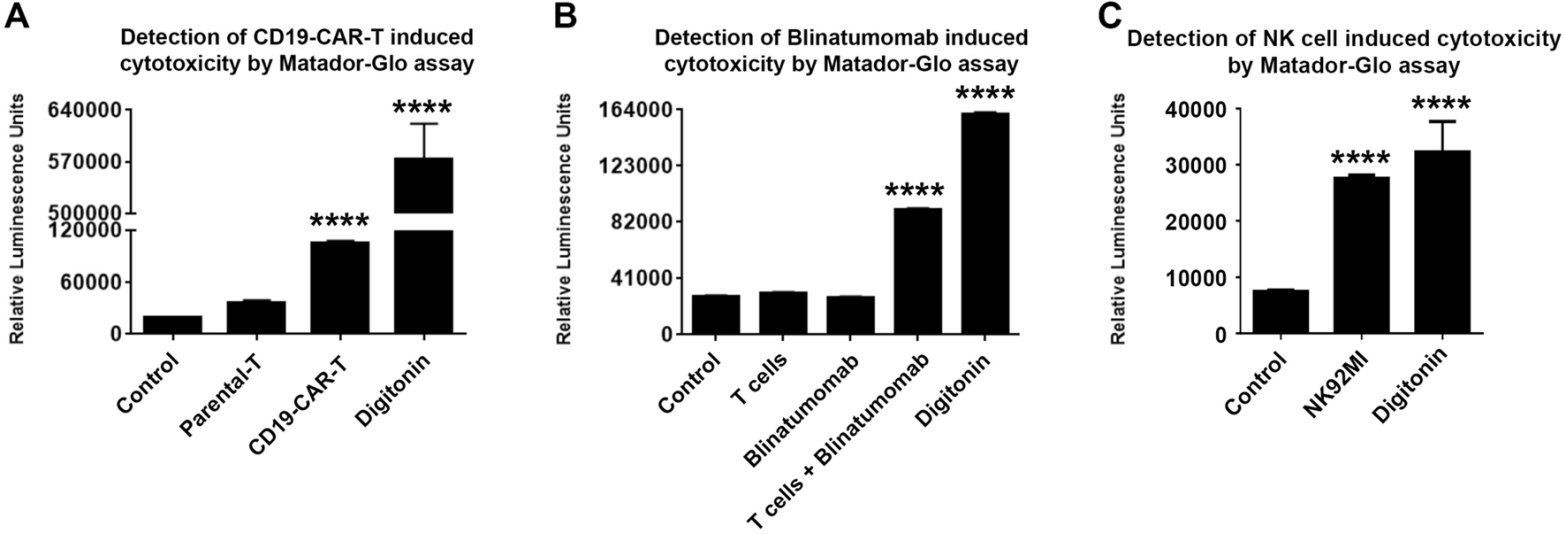
Applications of Matador-Glo assay in testing cytotoxicity induced by Immunotherapies. (**A**) Nalm6 cells stably expressing Luc146-1H2 were co-cultured in a 24-well plate at an E:T ratio of 1:5 for 48 h with Parental-T cells or T cells expressing the CD19-CAR in 1 ml total volume. Post-incubation, the cell homogenates were collected in 1.5 ml tubes, and 20 μl of cell homogenate was plated along with 40 μl of culture medium in a new 384-well plate in triplicate. Luminescence was detected by the addition of 15 µl of D-luciferin-containing assay buffer directly to each well. For maximum cell death Nalm6-Luc146-1H2 cells were treated with digitonin (30 µg/ml) for 60 min or PBS (control). The values shown are mean ± SE of a representative experiment performed in triplicate. (**B**) Primary human T cells were treated with Blinatumomab at a concentration of 100 ng/10^6^ cells/ml or vehicle (control) followed by co-incubation with Raji cells stably expressing Luc146-1H2 at an E:T ratio of 10:1 for 4 h. Luminescence was detected by the addition of D-luciferin-containing assay buffer directly to each well. The values shown are mean ± SE of a representative experiment performed in triplicate. (**C**) K562 cells stably expressing Luc146-1H2 were co-cultured with NK92MI cells in a 384-well plate at an E:T ratio of 1:1 for 4 h. Luminescence was detected as described above in (**A**). The values shown are mean ± SE of a representative experiment performed in triplicate. Statistically significant differences are shown by asterisks (****) at a level of *P* < 0.0001.

Cytotoxic assays are frequently used in the fields of cellular- and immune-therapy to test the cytotoxicity of chimeric antigen receptor (CAR)-expressing T cells, Bispecific T cell Engagers and antibody–drug conjugates. To test the utility of the Matador-Glo assay to measure CAR-T induced cytotoxicity, we generated primary human T cells transduced with a second generation CAR targeting CD19 based on a single chain variable fragment of the FMC63 antibody (CD19-CAR-T). Co-culture of CD19 positive Nalm6 cells stably expressing Luc146-1H2 with CD19-CAR-T resulted in a significant increase in luminescence activity, as compared to the parental primary human T cells (Parental-T) (Fig. 4A). We then tested the ability of the Matador-Glo assay to measure Blinatumomab (a BiTE expressing binding sites for both CD3 and CD19) induced cytotoxicity. As shown in Fig. 4B, co-culture of Raji cells stably expressing Luc146-1H2 with T-cells in the presence of Blinatumomab led to a sharp increase in the luminescence activity as compared to Raji-Luc146-1H2 cells treated with parental T-cells or Blinatumomab alone. We also tested the ability of Matador-Glo assay to measure natural killer cell (NK92MI) induced cytotoxicity. Co-culture of K562 cells stably expressing Luc146-1H2 with NK92MI for 4 h resulted in a significant increase in the luminescence activity as compared to control wells (media alone) (Fig. 4C). Digitonin was used as a positive control to maximum cell death (Fig. 4A–C). These results demonstrate the applications of Matador-Glo assay to detect cell death induced by immunotherapies.

### Luc146-1H2 is highly stable in cell culture medium

**Figure 5:**
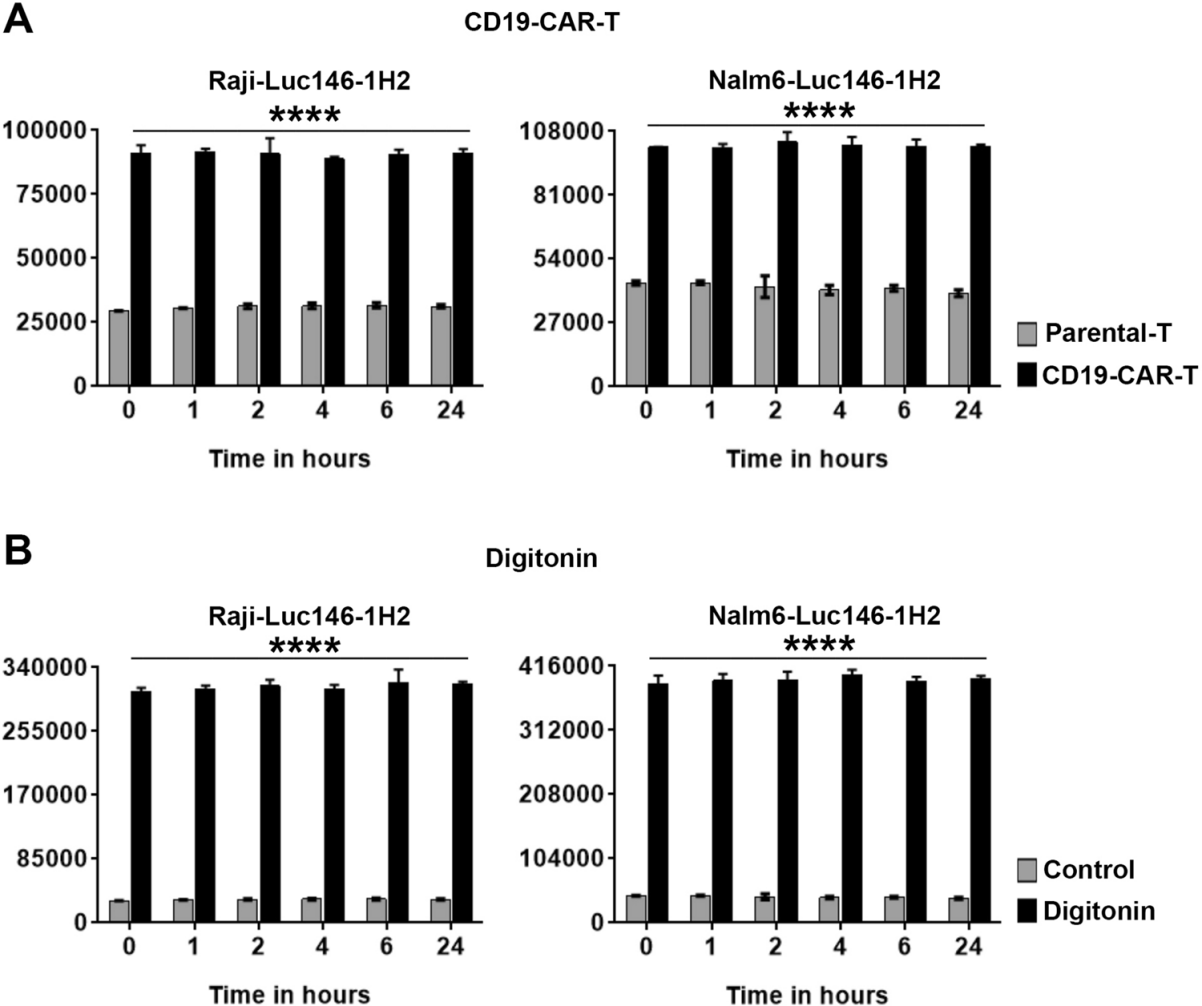
Luc146-1H2 is highly stable in cell culture medium. (**A**) Raji/Nalm6 cells stably expressing Luc146-1H2 were plated in a 24-well plate, co-cultured with parental-T cells or T cells expressing the CD19-CAR (CD19-CAR-T) at an Effector:Target (E:T) ratio of 1:1 for 18 h. After incubation, cell free supernatants were transferred into 6 different tubes and frozen immediately at − 80 °C. The tubes were directly transferred to 37 °C (from − 80 °C) and were incubated for indicated time periods (0–24 h). The luminescence was measured by adding D-luciferin containing assay buffer directly to each well. The values shown are mean ± SE of a representative experiment performed in duplicate for at least three times. (**B**) Raji/Nalm6 cells stably expressing Luc146-1H2 were plated in a 24-well plate, treated with media alone (control) or digitonin (30 μg/ml) for 90 min. After incubation, cell free supernatants were transferred into 6 different tubes and assayed as described above for (**A**). The values shown are mean ± SE of a representative experiment performed in duplicate for at least three times. Statistically significant differences are shown by asterisks (****) at a level of *P* < 0.0001.

Fluc has a very short half-life (< 30 min) in cell culture medium, which precludes its use in luciferase release-based cytotoxicity assays^8,12^. Luc146-1H2 is a thermostable luciferase that was developed by mutating the natural coding sequences of *LucPpe*^10^*.* Although Luc146-1H2 is considered thermostable, we wanted to confirm its stability under the assay conditions since proteases released during cell death may decrease its stability. Therefore, to test the stability of Luc146-1H2 in our assay, Raji and Nalm6 cells, stably expressing Luc146-1H2, were treated with a CD19-CAR-T at an E:T ratio of 1:1 for 18 h. After incubation, the cell-free supernatants were equally divided into 6 different tubes and frozen at − 80 °C. Next day, the tubes were placed at 37 °C for 0, 1, 2, 4, 6 and 24 h, followed by the measurement of luminescence activity using D-luciferin containing assay buffer. We observed significant increase in luminescence activity in wells that contained supernatants from CD19-CAR-T cells co-cultured with Raji or Nalm6 cells stably expressing Luc146-1H2 (Fig. 5A). More importantly, the luminescence values were stable for 24 h at 37 °C in cell culture supernatants (Fig. 5A). Similar results were obtained with supernatants from digitonin-treated Raji and Nalm6 cells stably expressing Luc146-1H2 (Fig. 5B). Collectively, these results demonstrate that Luc146-1H2 is highly stable in cell culture medium and therefore is suitable for luciferase release-based cell death assays.

### Matador-Glo assay can be coupled with Matador assay to develop a dual-luciferase cytotoxicity assay

**Figure 6:**
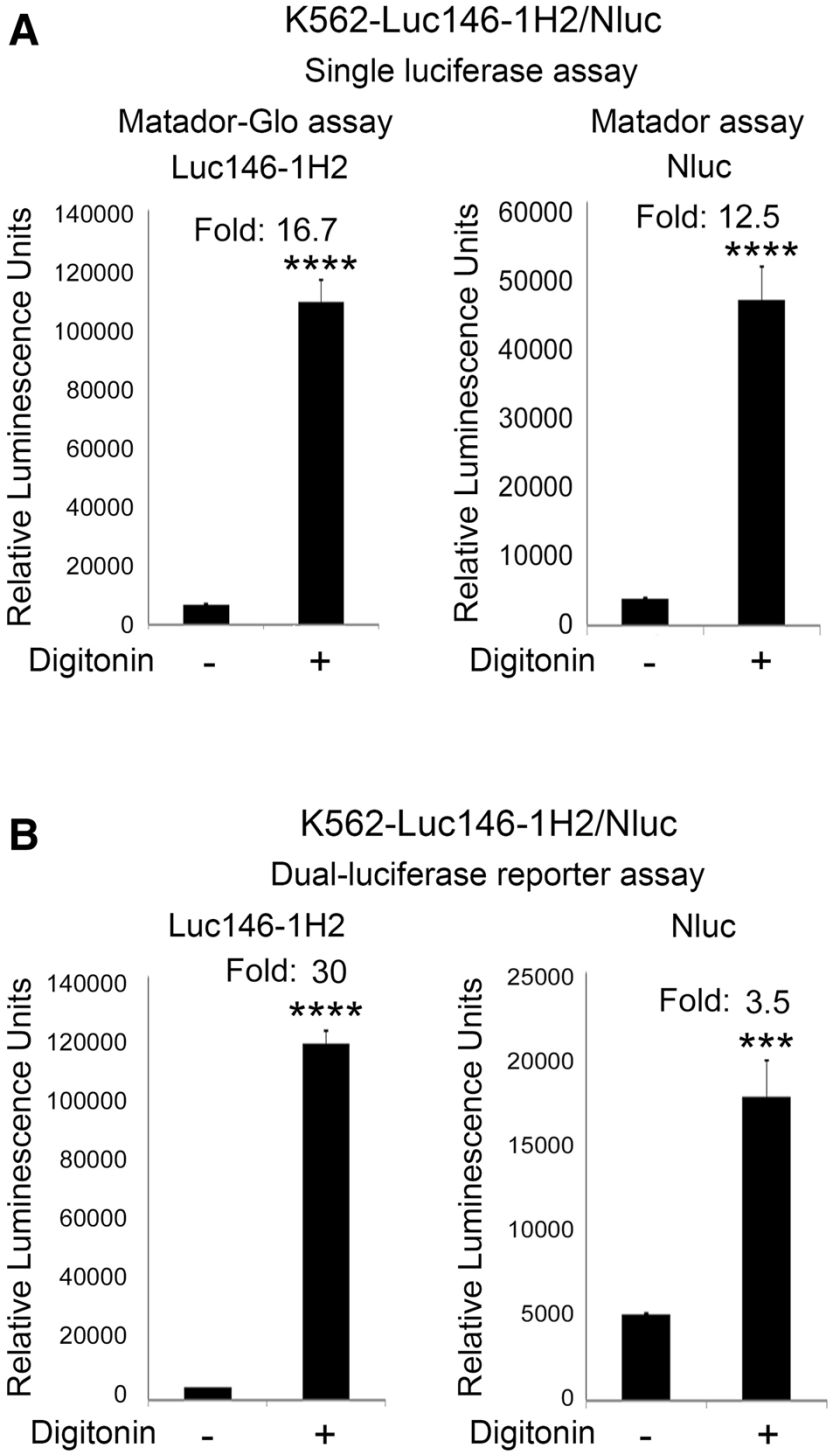
Matador-Glo assay can be coupled with Matador assay. (**A**) K562 cells stably expressing both Luc146-1H2 and Nluc (K562-Luc146-1H2/Nluc) were treated with digitonin (30 µg/ml) for 90 min. Post-incubation supernatants was carefully transferred to a 384-well plate to measure Luc146-1H2 activity using D-luciferin as a substrate (Matador-Glo assay) and Nluc activity using native coelenterazine as a substrate (Matador assay), respectively. (**B**) Dual-Luciferase Reporter assay kit from Promega (E1910) was used to measure samples from the same experiment as (**A**). Luc146-1H2 activity was measured first by adding Luciferase Assay Reagent II (LAR II). After quantifying the Luc146-1H2 activity, the Nluc activity was measured by adding Stop & Glo reagent to the same wells. The Stop & Glo Reagent both quenches the Luc146-1H2 signal and initiates the Nluc luminescence. The values shown are mean ± SE of a representative experiment performed in triplicate for at least two times. Statistically significant differences are shown by asterisks (***) at a level of *P* < 0.001 and (****) at a level of *P* < 0.0001.

Dual-luciferase assay systems have been described in the literature that can measure the activity of two luciferases sequentially from a single sample. The use of two different assay formats on a single sample allow the development of orthogonal assay with improved sensitivity, specificity and positive predictive value. They also help to reduce false negative results caused by the presence of luciferase inhibitors in the assay conditions as most such inhibitors are unlikely to inhibit both luciferases. The Matador-Glo assay described above uses D-luciferin assay buffer for luminescence detection, whereas the original Matador assay utilizes coelenterazine (CTZ). Therefore, we next asked the question if a dual-luciferase cytotoxicity assay can be developed by combining the Matador-Glo assay with the Matador assay. Dual-luciferase assays require luciferase inhibitors which inhibit or quench the activity of a first luciferase, allowing the measurement of the specific activity of second luciferase. In preliminary studies, we found that Luc146-1H2 activity can be inhibited by known luciferase inhibitors^14–16^, (Supplementary Fig. 1) thereby meeting this requirement. To develop a dual-luciferase based cytotoxicity assay, we generated K562 cells stably expressing both Luc146-1H2 and Nluc. K562-Luc146-1H2/Nluc cells were treated with digitonin (30 µg/ml) for 90 min. Post-incubation, cell homogenates were carefully transferred to a 384-well plate in three different sets. Samples from sets 1 and 2 were used to measure Luc146-1H2 and Nluc activities using D-Luciferin and CTZ assay buffers, respectively. As shown in Fig. 6A, treatment of K562-Luc146-1H2/Nluc cells with digitonin resulted in 16.7- and 12.5-fold increase in Luc146-1H2 and Nluc luciferase activities when measured individually in separate wells by addition of D-Luciferin and coelenterazine assay buffers, respectively. The samples in set 3 were analyzed using a dual-luciferase reporter assay kit from Promega. For this purpose, the Luc146-1H2 activity was measured first, followed by the addition of stop-and-glo reagent which quenches the Luc146-1H2 activtiy and stimulates Nluc activity. As shown in Fig. 6B, treatment with digitonin resulted in a 30-fold and 3.5-fold increase in Luc146-1H2and Nluc activities, respectively, when measured using the dual-luciferase assay kit. Taken collectively, these results suggest that the Matador-Glo and Matador assay can be combined to develop a dual-luciferase cytotoxicity assay which allows measurement of two different luciferases from a single sample.

### Development of a multiplex Matador-Glo/Matador assay to measure on-target and off-target cytotoxicities

**Figure 7:**
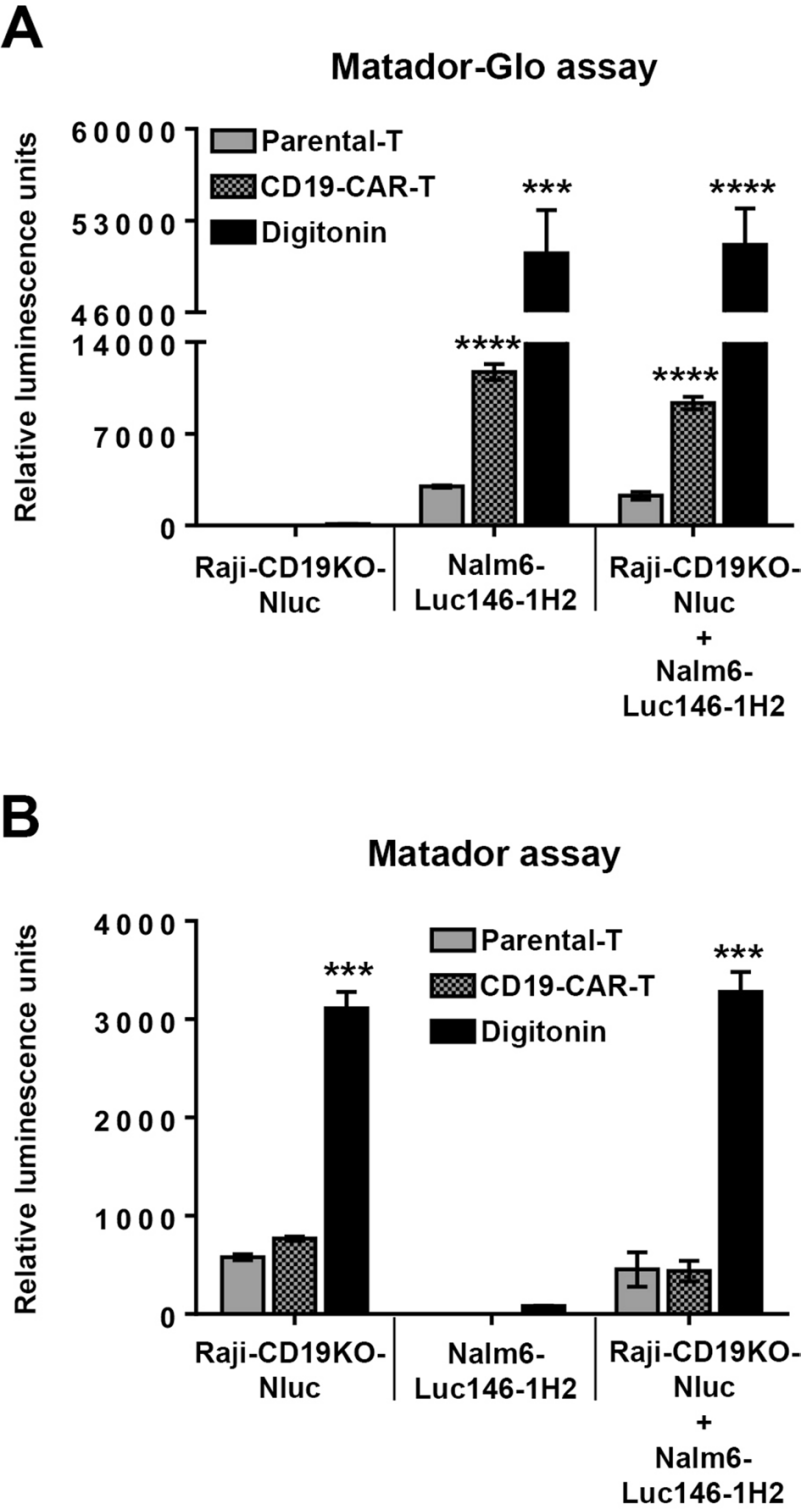
Utility of the Matador-Glo assay in a multiplex cytotoxicity assay. (**A**) Raji cells lacking CD19 expression and stably expressing Nluc (Raji-CD19KO-Nluc) and CD19-positive Nalm6 cells stably expressing Luc146-1H2 (Nalm6-Luc146-1H2) were either plated alone or in combination at 1:1 ratio. The plated cells were co-incubated with parental-T cells or T cells expressing the CD19-CAR (CD19-CAR-T) at an Effector:Target (E:T) ratio of 1:1 for 24 h in a 24-well plate. For maximum cell death, 30 µg/ml digitonin was added an hour prior to luciferase assay. Post-incubation cell homogenates were carefully transferred to a 384-well plate to measure Luc146-1H2 activity (Matador-Glo assay) using D-luciferin containing assay buffer. (**B**) The same samples mentioned in (**A**) were used to measure Nluc activity (Matador assay) using coelenterazine containing assay buffer. The values shown are mean ± SE of a representative experiment performed in triplicate for at least two times. Statistically significant differences are shown by asterisks (***) at a level of *P* < 0.001 and (****) at a level of *P* < 0.0001.

In the preceding section, we demonstrated that it is possible to develop a dual/multiplex cytotoxicity assay by combining the Matador-Glo and Matador assays using a target cell line co-expressing both Luc146-1H2 and Nluc. An ideal immunotherapeutic agent (e.g., CAR-T) should demonstrate specific cytotoxicity against cells that express its target antigen and show negligible non-specific cytotoxicity against cells that lack expression of its target antigen. This necessitates setting up two different co-culture cytotoxicity experiments; i.e., with antigen-positive and antigen-negative cells. Thus, a CD19-CAR-T product is generally tested against a CD19-expressing cell line (e.g., Raji or Nalm6) and a CD19-negative cell line (e.g., HL60 or Raji-CD19-KO). We wanted to test if the Matador-Glo assay can be combined with the Matador assay to test cytotoxicity against antigen-positive and antigen-negative cell lines in a single experiment. For this purpose, a clone of Raji cells lacking the expression of CD19 was generated using CRISPR/Cas9 technology which was subsequently engineered to stably express Nluc (Raji-CD19KO-Nluc) and served as the antigen-negative target cell line^17^. Nalm6 cells stably expressing Luc146-1H2 (Nalm6-Luc146-1H2) served as the antigen-positive target cell line. The two target cell lines were either plated alone or mixed 1:1 and then co-cultured with either parental-T cells or CD19-CAR-T cells at a 1:1 (Effector:Target) ratio in a 24-well plate for 24 h. Digitonin-treated wells were used as a positive control for cell death. Post-incubation, cell suspensions were carefully transferred to a 384-well plate in two sets. Samples from sets 1 were used to measure Luc146-1H2 following the addition of D-luciferin assay buffer (Fig. 7A), while samples from set 2 were used to measure Nluc activity following the addition of CTZ assay buffer (Fig. 7B). As shown in Fig. 7A, when samples from set 1 were read for luminescence using the D-luciferin assay buffer, luminescence signal was detected only in the wells that contained Nalm6-Luc146-1H2 cells either alone or in combination with Raji-CD19KO-Nluc. More importantly, the magnitude of luminescence signal in wells containing only the Nalm6-Luc146-1H2 cells was similar to the signal in wells containing both Nalm6-Luc146-1H2 and Raji-CD19KO-Nluc cells, thereby demonstrating that the presence of Raji-CD19KO-Nluc cells did not interfere with the signal derived from Nalm6-Luc146-1H2 cells. Similarly, when samples from set 2 were read for luminescence using the CTZ assay buffer, luminescence signal was detected only in the wells that had Raji-CD19KO-Nluc either alone or in combination with Nalm6-Luc146-1H2 cells (Fig. 7B). Again, the magnitude of Nluc signal in wells containing only the Raji-CD19KO-Nluc cells was similar to the signal in wells containing both Raji-CD19KO-Nluc cells and Nalm6-Luc146-1H2 cell, thereby demonstrating that the presence of Nalm6-Luc146-1H2 cells had no significant adverse effect on the magnitude of signal derived from Raji-CD19KO-Nluc cells. Collectively, these results demonstrate lack of substrate cross-reactivity between Luc146-1H2 and Nluc, thereby allowing target specific cytotoxicity measurement within a mixture of cells expressing these luciferases in a single experiment.

### Use of the Luc146-1H2 expressing cells for in vivo imaging

**Figure 8:**
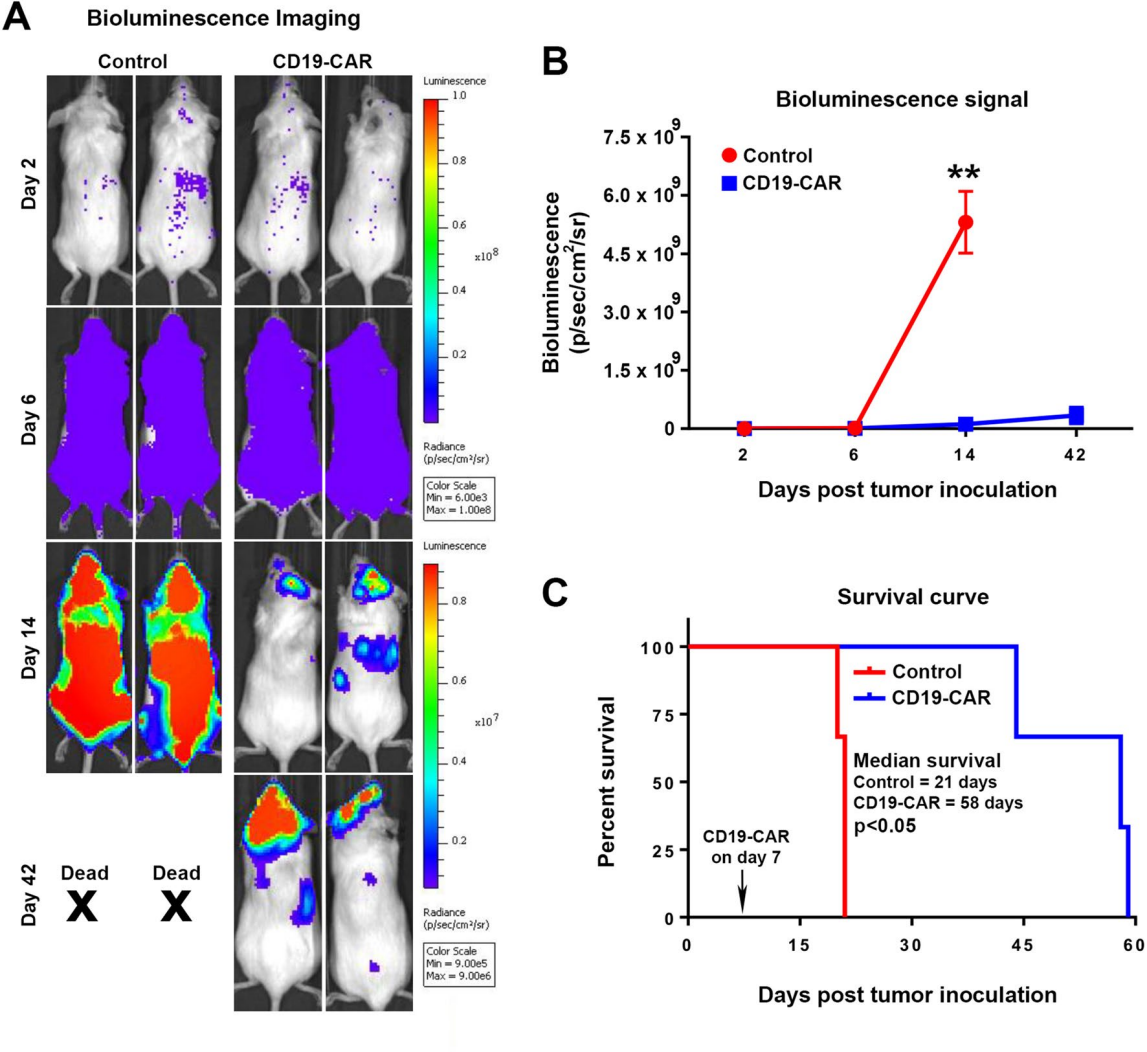
Utilizing Luc146-1H2 expressing target cells for in vivo experiments. (**A**) 6-week-old NOD.Scid-Gamma (NSG) mice were injected with one million Nam6-Luc146-1H2 cells via tail vein. Bio-Luminescence Imaging (BLI) was performed on days 2 and 6 post injection to confirm the engraftment of tumor cells (Nam6-Luc146-1H2) in mice. Post confirmation, on day 7 mice were randomly divided into control (parental-T cells) and CD19-CAR (3 × 10^6^ CAR positive cells) treatment groups and were injected with respective cells via tail vein. Representative serial BLI images of mice on days 2, 6, 14 and 42 of indicated groups are shown. (**B**) Tumor burden, as measured by relative luminescence measurements of serial BLI imaging from control and CD19-CAR-T treated mice. (**C**) Survival curves (Kaplan–Meier) of mice bearing Nalm6-Luc146-1H2 cells treated with parental-T cells (control) or CD19-CAR-T cells are shown (n = 3 in each group). The survival curve was generated in GraphPad Prism 5 software. Statistically significant differences are shown by asterisks (**) at a level of *P* < 0.01.

One of the major limitations of the original Matador assay is that cell lines engineered to stably express marine luciferase can not be used for i*n vivo* bioluminescence imaging applications due to high background and cost of coelenterazine. Therefore, we next tested whether Luc146-1H2 expressing cells generated for Matador-Glo assay can be also used for in vivo bioluminescence imaging. For this purpose, we injected 1 million Nalm6-Luc146-1H2 cells by intravenous injection in the tail vein of immune-deficient NSG mice. Seven days after the injection, animals were randomly assigned to two separate groups. Mice from group 1 were injected with 3 million parental-T cells (control), while mice from group 2 received equal number of CD19-CAR-T cells. Bioluminescent (BLI) images were obtained on days 2, 6, 14, and 42 post target cells injection. As shown in Fig. 8A, B, the two groups of mice had similar leukemia burden before the injection of CD19-CAR-T cells (days 2 and 6). A significant decrease in BLI signal was observed in the mice that were treated with CD19-CAR-T (day 14), indicating a reduction in leukemia burden (Fig. 8A, B). In line with the BLI data, Nalm6-Luc146-1H2 bearing mice that were injected with parental-T cells (control) died by day 21 (median survival = 21 days) due to leukemia (Fig. 8C). In contrast, there was a marked increase in the median survival of Nalm6-Luc146-1H2 bearing mice that were treated with CD19-CAR-T cells (median survival = 58 days) (Fig. 8C). These results indicate the Luc146-1H2 expressing target cells that were engineered for the Matador-Glo assay can also be used for in vivo serial BLI imaging applications, thereby saving significant time, labor and resources. Finally, we also found that ectopic expression of Luc146-1H2 did not alter the sensitivity of cell lines to several targeted small molecule inhibitors of cell growth (Supplementary Fig. 2), thereby indicating that Luc146-1H2 expressing cells can also be used for in vivo BLI imaging studies involving small molecule inhibitors^18–22^.

## Discussion

In recent years, significant progress has been made in the utilization of immunotherapeutic approaches for the treatment of cancer. These immunotherapeutic approaches include CARs and Bispecific T cell engagers that can target specific Tumor-Associated Antigens (TAA)^23^. A cytotoxicity assay is a critical experimental test for testing of any novel immunotherapeutic approach in cancer. Furthermore, cytotoxicity assays are needed as potency assays and product-release assays during the manufacturing of cell therapy products.

In the field of immunotherapy, there is a need for an efficient universal method to measure target cell death. Attempts have been made by us and others to develop a Fluc-release based cytotoxicity assay^8,12,13^. However, the short half-life of Fluc (< 30 min) in cell-culture medium and inconsistency in cell death detection has hampered its successful use in a cytotoxicity assay^8,12,13^. Recently, we described a marine luciferase release-based cytotoxicity assay, termed Matador Assay, that involves the expression of a marine luciferase in the target cells^8^. The Matador assay is highly sensitive, specific, rapid, and can be performed in a single-step manner without the need for any expensive equipment^8^. The Matador assay uses coelenterazine as a substrate which is relatively expensive as compared to D-Luciferin, the substrate for Fluc. An additional limitation of marine luciferases is the high background when used for in vivo bioluminescence imaging applications. This has precluded the use of marine luciferase expressing cell lines generated for Matador assay for in vivo bioluminescence applications and has necessitated the generation of a separate panel of cell lines expressing Fluc for this purpose. Thus, neither marine luciferases nor Fluc are ideal for both in vitro and in vivo testing for novel immunotherapeutic agents.

To overcome the above limitation of marine luciferases and Fluc, we took advantage of a thermostable luciferase (Luc146-1H2) that uses D-luciferin as a substrate, is stable in culture medium and can be used for in vivo bioluminescence imaging. We demonstrate that similar to the marine luciferase-based Matador assay, the thermostable Luc146-1H2-based Matador-Glo assay has several advantages over the other cytotoxicity assays. First, the assay is highly sensitive and is capable of detecting cell death at a single cell level in some cell lines. Second, unlike the radio-active chromium release or fluorescent dye release assays, there is no need to preload the target cells on the day of the experiment or to perform multiple washes to remove the excess label, thereby saving cost, time, and labor. Third, the Matador-Glo assay can be performed as a one-step homogeneous assay without the need for a multistep assay format involving the separation of cells from the supernatant as is needed for other cytotoxicity assays such as Cr^51^, LDH, and Fluorescent dye release-based assays. Fourth, the Luc146-1H2 used in the Matador-Glo assay is highly stable in cell culture media (> 24 h), which makes it suitable for testing the cytotoxicity of most immunotherapies that are tested in assays with durations of 4 h or more.

The Matador-Glo assay also has several advantages over the original Matador assay. Matador-Glo is a cost-effective assay because D-luciferin, the substrate for Luc146-1H2 used in the Matador-Glo assay, is very cheap and widely available in comparison to coelenterazine, the substrate for marine luciferases used in the Matador assay. Furthermore, we demonstrate that both Luc146-1H2 and a marine luciferase (e.g., Nluc) can be simultaneously expressed in a single cell line and such dual luciferase expressing cell line can be used to develop orthogonal and dual-luciferase cytotoxicity assays that can measure increases in the activity of two different luciferase from a single experiment. Finally, we demonstrate that it is also possible to separately express Luc146-1H2 and a marine luciferase in two different cell lines (e.g., a test and a control cell line or an antigen-positive and an antigen-negative cell line) and then combine the two cell lines in a single well with an immunotherapeutic agent (e.g., a CAR-T product or Bispecific T cell engager) to measure its cytotoxicity against both cell lines simultaneously. In addition to reducing cost and improving throughput, such a format would be appealing in case the immunotherapeutic agent is limiting in amount.

We further demonstrate that Luc146-1H2 expressing cells generated for use in the in vitro cytotoxicity testing using Matador-Glo assay can be used for *in viv*o testing involving serial Bio-Luminescence Imaging (BLI), thereby potentially decreasing the amount of time and labor involved in generating stable cell lines expressing Fluc for in vivo studies. In fact, our results presented in Fig. 8, clearly demonstrate the ability to use Luc146-1H2 expressing Nalm6 cells, which were generated for use in the Matador-Glo assay, for in vivo efficacy testing of CD19-specific CAR-T cells by serial BLI imaging. We have also generated many additional Luc146-1H2 expressing target cells and used them for in vivo testing in our pre-clinical cellular therapy studies. As of now, we have not come across any cell line in which the Luc146-1H2 cells did not work as expected in the in vivo BLI experiments. Therefore, Luc146-1H2 has become our choice of luciferase for expression in target cells for the purpose of testing novel cell and immune therapies. Luc146-1H2 is a thermostable variant created by mutating the naturally occurring beetle luciferase (*LucPpe).* Multiple studies have shown that specific mutations in luciferases will shift their emission spectrum, therefore, further studies are needed to check whether the Luc146-1H2 mutant would led to a change in emission spectrum in comparison to *LucPpe* that can be potentially exploited for multicolor in vivo BLI imaging^24–27^.

## Materials and methods

### Construction of lentiviral-based expression vectors

The construct expressing Nluc has been described previously^8^. A gene fragment encoding a thermostable mutant of *LucPpe* (Luc146-1H2) was codon-optimized and synthesized by Integrated DNA Technologies (IDT). The gene fragment was used as a template in PCR reaction using custom primers and then cloned in a lentiviral expression vector (pLenti-EF1α) using standard molecular biology techniques. The construct also co-expressed a puromycin resistance gene in frame with the cDNAs encoding Luc146-1H2 and separated from it by a 2A ribosomal skip sequence. The pLenti-blast vector was derived from pLenti6v5gw_lacz vector (Invitrogen; Thermo Fisher Scientific) by removal of the lacz gene. psPAX2 was a gift from Didier Trono (Addgene plasmid # 12,260). The pLP/VSVG envelope plasmid was obtained from Invitrogen (Thermo Fisher Scientific). A CD19-specific CAR construct was developed as described in our previous studies^8,17,28^.

### Cell lines and reagents

Cell lines were maintained and cultured as described in our previous studies^8,17,28,29^. K562 (Chronic Myelogenous Leukemia), Raji (Burkitt’s lymphoma), and NK92MI (Natural killer cell) cell lines were obtained from ATCC and maintained as per the instructions provided. Nalm6 (Acute Lymphoblastic Leukemia), BC-3 (Primary Effusion Lymphoma), MM1.S (Multiple Myeloma) and U266 (Multiple Myeloma) were kindly provided by Dr. Markus Muschen, Dr. Jae Jung, Dr. Alan Lichenstein and Dr. Gregor Adams, respectively. 293FT cells were obtained from Invitrogen (Thermo Fisher Scientific) and cultured as recommended. Peripheral blood mononuclear cells (PBMCs) were obtained and utilized as described in our previous studies^8,17,28^. PBMCs were isolated by the Ficoll gradient method from platelet depleted donor cells obtained from the Blood Bank at Children Hospital of Los Angeles (CHLA). PBMCs were subsequently used to isolate T cells using CD3 magnetic microbeads (Miltenyi Biotech) following the manufacturer’s instructions. T cells were cultured in XVIVO-15 (Lonza) medium supplemented with 100 IU/ml recombinant human IL2 and 30 ng/ml soluble antibodies to human CD3 and CD28. All the cells were cultured at 37 °C in a 5% CO2 humidified incubator. Reagents were made and utilized as described in our previous studies^8,17,28^. Blinatumomab (BiTE) was obtained from Amgen. Digitonin, polybrene, ATP, DTT, and Resveratrol were obtained from Sigma. Linear polyethylenimine (PEI) was purchased from Polysciences. Native coelenterazine was purchased from Nanolight technology. D-luciferin was obtained from GoldBio. Luciferase Inhibitor I was obtained from Millipore. PTC-124 was obtained from BioVision.

### Preparation of lentiviral supernatants

Lentiviruses were generated in 293FT cells as described in our previous studies^8,17,28^. For lentivirus production, 10 µg (μg) of lentiviral expression plasmid was used along with 6 μg of PSPAX2 and 3 μg of PLP/VSVG plasmid as packaging vector for a 100 mm plate (containing 10 ml media) using the PEI method. 48 h post-transfection, the media containing lentivirus was collected, filtered (using 0.45-micron syringe filter), and used to transduce cells.

### Transient transfection of 293FT cells with lentiviral-based luciferase expression vectors

The 293FT cells were transfected in a 24-well plate with various expression plasmids (500 ng/well) using the PEI method as described previously^17^. Approximately 24 h post-transfection, the cells were either left untreated or treated with 30 μg/ml digitonin to induce cell death, followed by the detection of luminescence as described under luciferase assays^8^.

### Generation of stable cancer cell lines expressing various luciferases

Stable cell lines expressing the different luciferase reporters were generated as described previously^8^. Approximately 0.5 × 10^6^ cells in 1 ml of medium was infected with 2 ml of viral supernatant in the presence of polybrene (8 μg/ml) in a 6-well plate by spin-infection (1800 rpm for 45 min at 32 °C) followed by incubation of these plates at 37 °C in a CO_2_ incubator overnight. Next morning, the cells were pelleted and re-suspended in media with their respective antibiotics for selection of stably transduced cells^8^.

### Infection of primary human T-cells with chimeric antigen receptors

Second generation CAR constructs were used to infect primary human T cells. The lentiviral supernatants were concentrated by centrifugation at 18,500 rpm for 2 h at 4 °C. The viral pellets were re-suspended in 1/10 of the initial volume and stored at − 80 °C. In general, primary T cells were infected using spin-infection in the presence of polybrene essentially as described previously^8,17,28^. The media was changed 6 h after spinfection. The infection was repeated for two more days for a total of 3 infections. After the 3^rd^ infection, the cells were pelleted and re-suspended in complete T-cell media^8^.

### Luciferase assays

Coelenterazine (CTZ) was used as a substrate to measure luminescence in cells expressing Nluc, using a CTZ-assay buffer containing 20 μM CTZ in PBS^30^. CTZ stock solution (2 mM) was made with acidified methanol as recommended by the manufacturer. Luc146-1H2 activity was determined using the Fluc assay buffer (1 mM D-luciferin, 25 mM Gly-Gly, 15 mM potassium phosphate, 15 mM MgSO_4,_ 4 mM EGTA, 2 mM ATP, and 1 mM DTT) as described earlier^31^. Unless indicated otherwise, the assay buffer with substrate was added to cell culture media at 1:1 or 1:4 ratio (v/v) for all luciferase assays. Luminescence was read in endpoint mode using BioTek synergy H4 hybrid microplate reader for 10 s^8^.

### Generation of K562 cells co-expressing Luc146-1H2 and Nluc and dual luciferase assays

K562 cells stably expressing plenti-Luc146-1H2-Pac were infected with plenti-Nluc-blast lentiviral supernatant followed by selection with blasticidin. The cells were used to develop dual luciferase assays using the Dual-luciferase reporter assay kit from Promega (E1910). Briefly, Luc146-1H2 activity was measured first by adding Luciferase Assay Reagent II (LAR II). After quantifying the Luc146-1H2 activity, the Nluc activity was measured by adding Stop & Glo reagent to the same wells. The Stop & Glo Reagent both quenches the Luc146-1H2 signal and initiates the Nluc luminescence.

### Animal experiments

A total of 1 million Nalm6 cells stably expressing Luc146-1H2 (Nalm6-Luc146-1H2) in 100 μL of PBS was injected into approximately 6-week-old female NOD.Scid-Gamma (NSG) mice (Jackson laboratory) via tail vein. To assess the presence of tumor cells, mice were imaged on days 2 and 6 post-inoculation using an IVIS spectrum imaging system (Perkin Elmer’s Waltham, MA, USA). Seven days after tumor cells inoculation, mice were randomly divided into two groups and were injected via tail vein with 3 million CD19-CAR positive T cells (CD19-CAR group) or corresponding number of parental T cells (control group). The animals were monitored for survival and tumor progression was monitored by serial BLI imaging. All animal handling procedures were performed with the approval of the University of Southern California (USC) Institutional Animal Care and Use Committee (IACUC), in accordance with ethical guidelines and regulations, and complied with ARRIVE guidelines.

### Statistical analysis

Two-tailed unpaired Student *t* test was used to test for differences between 2 groups using GraphPad Prism 5 software. Differences with a *P* ≤ 0.05 were considered statistically significant.

## Supplementary Information

Supplementary Information 1.

## Data Availability

The data and reagents will be available upon request to senior author P.M.C.
