# Supplementary material for: A novel thermostable beetle luciferase based cytotoxicity assay
Source: Sci Rep. 2021 May 11;11:10002. doi: 10.1038/s41598-021-89404-z (PMC8113442; doi:10.1038/s41598-021-89404-z)
Supplement: Supplementary file 1 — Supplementary Information 1. [file 41598_2021_89404_MOESM1_ESM.docx]

**Supplementary Material**

**­ A novel thermostable beetle luciferase based cytotoxicity assay**

Sunju Choi**^*^**, Hittu Matta**^*^**, Ramakrishnan Gopalakrishnan**^*^**, Venkatesh Natarajan, Songjie Gong, Alberto Jeronimo, Wei-Ying Kuo, Bryant Bravo and Preet M. Chaudhary^#^

*These authors contributed equally to this work.

Jane Anne Nohl Division of Hematology and Center for the Study of Blood Diseases, University of Southern California, Keck School of Medicine, Los Angeles, California, United States of America.

^#^**Corresponding author:** Preet M. Chaudhary, M.D., Ph.D.

**
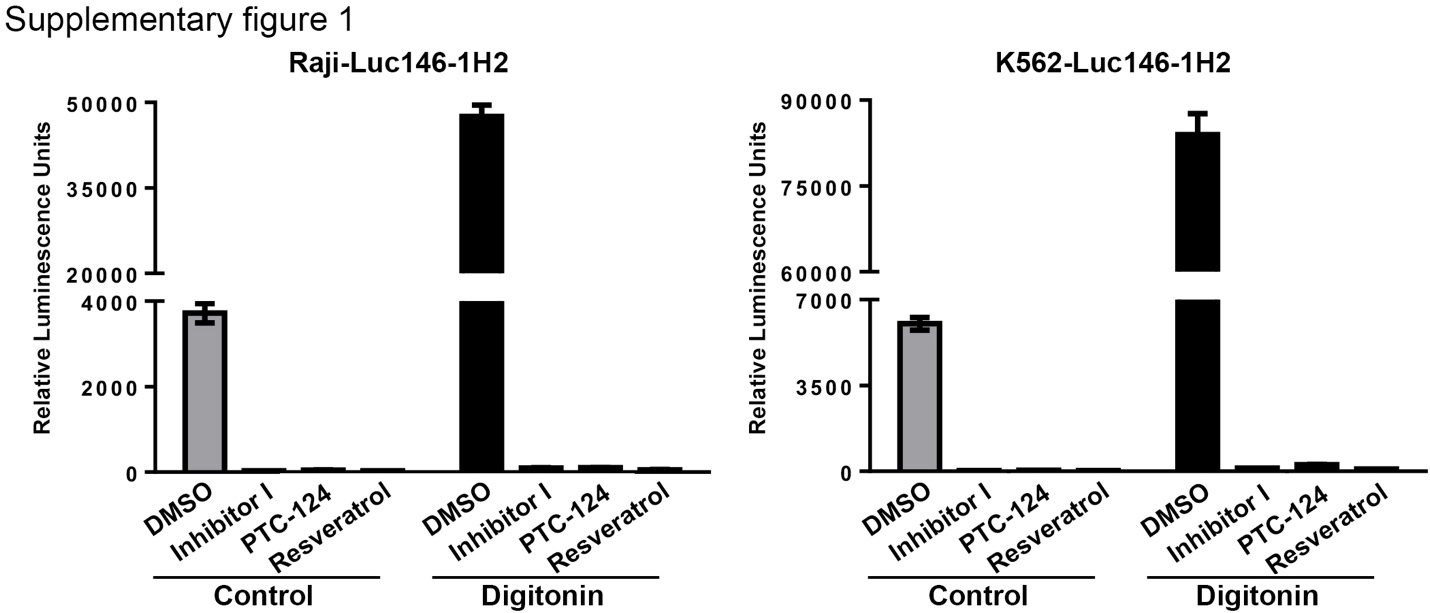
Supplementary Figure 1. Luc146-1H2 activity can be inhibited by luciferase inhibitors.** Raji/K562 cells stably expressing Luc146-1H2 were plated (30000 cells in 15 μl) in a 384-well lumitrac plate and treated with 15 μl of digitonin (30 µg/ml final concentration) for 90 minutes or 15 media control. Luciferase inhibitors were added in 10 μl volume (Luciferase inhibitor I: 20 μM; PTC-124: 25 μM; Resveratrol: 100 μM) 5 minutes before the read. The plate was read for luminescence by adding D-luciferin containing assay buffer (20 µl) directly to each well. The values shown are mean±SE of a representative experiment performed in triplicate.

**
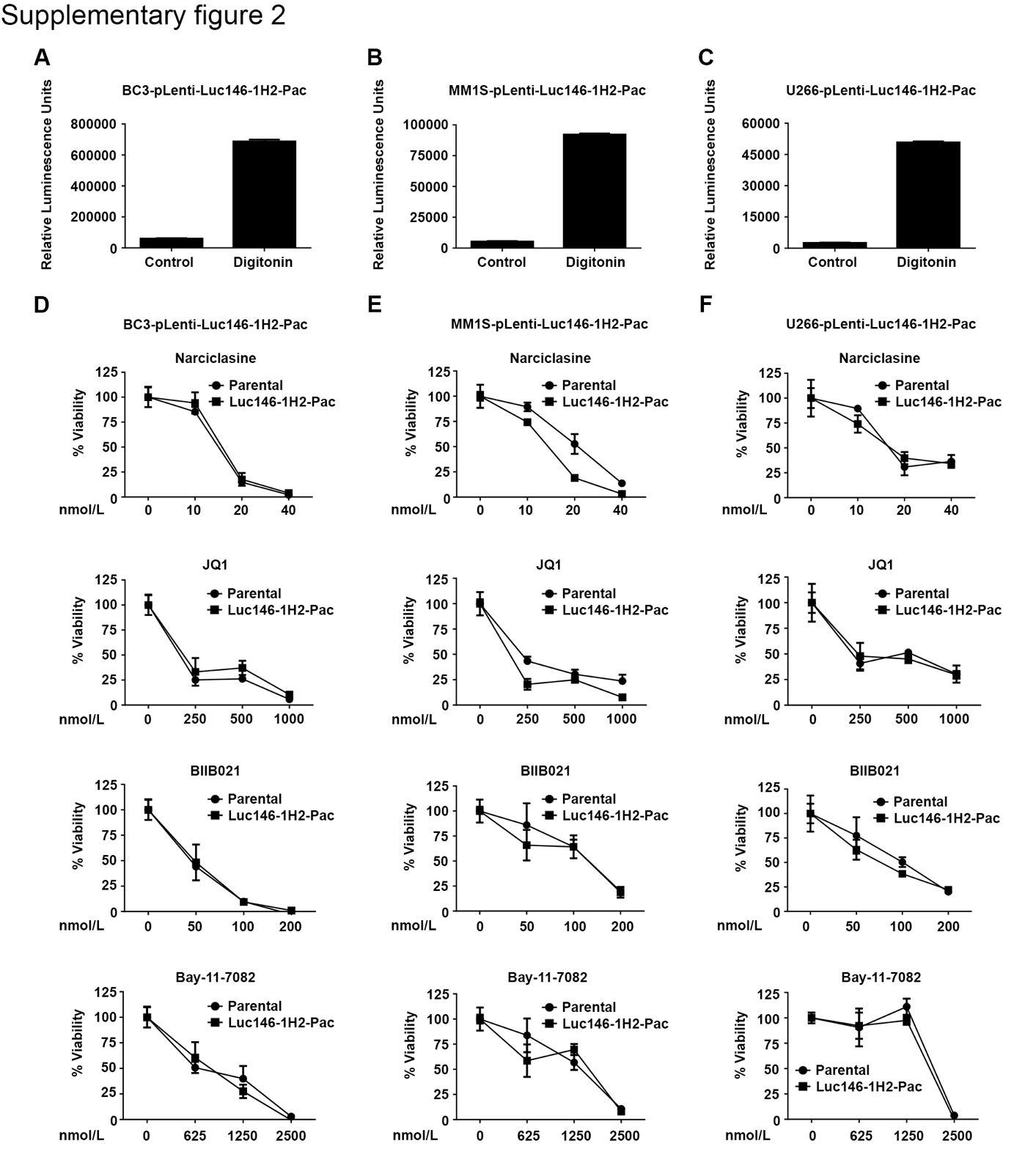
**

**Supplementary Figure 2. Ectopic expression of Luc146-1H2 did not alter the sensitivity of cell lines to small molecule inhibitors.** **(A-C)** Indicated cell lines stably expressing Luc146-1H2 were plated in a 384-well lumitrac plate and treated with digitonin (30 µg/ml) for 90 minutes or media control. The plate was read for luminescence by adding D-luciferin containing assay buffer (20 µl) directly to each well. **(B-F)** Indicated cell lines were treated with increasing concentrations of narciclasine, JQ-1, BIIB021, and Bay-11-7082 for 4 days. Cell viability was measured using an MTS (3-(4,5-dimethylthiazol-2-yl)-2,5-diphenyltetrazolium bromide) assay. The values shown are mean±SE of a representative experiment performed in triplicate.
